# Identification of Key Genes Related to Both Lipid Metabolism Disorders and Inflammation in MAFLD

**DOI:** 10.3390/biomedicines13092211

**Published:** 2025-09-09

**Authors:** Xin Dai, Yuhong Hu, Ke Zhang, Bangmao Wang, Jie Zhang, Hailong Cao

**Affiliations:** 1 Tianjin Key Laboratory of Digestive Diseases, National Key Clinical Specialty, Tianjin Institute of Digestive Diseases, Department of Gastroenterology and Hepatology, Tianjin Medical University General Hospital, Tianjin 300052, China; daixin1986@tmu.edu.cn (X.D.); YuhongHu09@tmu.edu.cn (Y.H.); mwang02@tmu.edu.cn (B.W.); 2 Department of Pharmacy, Tianjin Medical University General Hospital, Tianjin 300052, China

**Keywords:** lipid metabolism disorders, inflammation, metabolic-associated fatty liver disease, machine learning, single-cell sequencing

## Abstract

**Background:** Both lipid metabolism disorders and inflammation are critical contributors to the progression of metabolic-associated fatty liver disease (MAFLD), yet integrated analyses identifying key genes linking them remain scarce. **Methods:** Differentially expressed genes in MAFLD were extracted from the GSE135251 dataset and intersected with lipid metabolism- and inflammation-related genes from Molecular Signatures Database (MSigDB). Machine learning on GSE135251, followed by validation on GSE89632, identified key genes. Functional enrichment, immune microenvironment profiling, and nomogram analysis were subsequently conducted. Cellular heterogeneity was assessed using the single-cell sequencing (scRNA-seq) dataset GSE186328, and gene expression in MAFLD mice was validated via real-time Polymerase Chain Reaction (PCR). Activators targeting these genes were predicted using Drug Signatures Database (DsigDB). **Results:** Four genes—FADS1, FADS2, GLB1, and PNPLA3—were identified as key regulators involved in both lipid metabolism disorders and inflammation in MAFLD. These genes were co-enriched in ribosome-related pathways. GLB1 correlated strongly with CD56dim natural killer cells in immune infiltration analysis. A diagnostic nomogram integrating these genes demonstrated exceptional discriminatory power, with Area Under the Curve (AUC) values of 0.98981 for GSE135251 and 0.9204 for GSE89632. ScRNA-seq revealed elevated FADS1, FADS2, and GLB1 expression in MAFLD-associated NK/T cells compared to controls. Real-time PCR confirmed significant upregulation of all four genes in MAFLD mice. Drug prediction identified estradiol as a potential activator targeting these genes. **Conclusions:** This study identified FADS1, FADS2, GLB1, and PNPLA3 as key genes involved in the progression of MAFLD, linking metabolic dysfunction and inflammation.

## 1. Introduction

Metabolic-associated fatty liver disease (MAFLD), previously known as non-alcoholic fatty liver disease (NAFLD), can now also be referred to as Metabolic Dysfunction-Associated Steatotic Liver Disease (MASLD) since the term was proposed at the 2023 European Association for the Study of the Liver Congress. MAFLD is the most common chronic liver disease and the leading cause of elevated liver enzymes in populations undergoing health screening worldwide. Recent estimates indicate that approximately 38.77% of the global population is affected by MAFLD, with the incidence increasing by nearly 50% over the past three decades. As a manifestation of multi-system metabolic dysfunction involving the liver, MAFLD is often associated with type 2 diabetes, metabolic syndrome, hypertension, and coronary atherosclerotic heart disease, placing a significant burden on healthcare systems [1,2,3]. Consequently, MAFLD has emerged as a major global health challenge. It is of great importance to delve deeply into the mechanisms underlying MAFLD and to investigate potential treatments for it.

Lipid metabolic disorders and inflammation are two primary contributors to the progression of MAFLD. Lipid metabolism disorders are hallmarks of MAFLD and serve as primary drivers of disease progression [4]. The disease begins with the abnormal accumulation of lipids in hepatocytes, a process that triggers mitochondrial dysfunction and increases oxidative stress. Prolonged exposure of hepatocytes to high levels of lipid peroxidation and oxidative stress eventually leads to cell death due to lipotoxicity [5]. In parallel, damaged hepatocytes release danger signals that activate macrophages and other infiltrating inflammatory cells in the liver. These cells secrete pro-inflammatory cytokines, which exacerbate hepatocyte damage and death, contributing to the development of steatohepatitis. Chronic inflammation further accelerates liver damage and fibrosis, and can eventually lead to severe conditions such as cirrhosis and even hepatocellular carcinoma [6,7]. Despite these insights, research has mainly focused on identifying key genes involved in either lipid metabolism or inflammation separately, with few studies integrating both aspects.

The rapid development of next-generation sequencing technologies and bioinformatics approaches has significantly advanced our understanding of disease pathogenesis and the identification of potential therapeutic agents. Among these, machine learning algorithms have emerged as powerful tools for identifying key disease-related genes. In this study, we utilized bulk RNA sequencing and single-cell RNA sequencing (scRNA-seq) data from the Gene Expression Omnibus (GEO) database, combined with bioinformatics methods such as machine learning, to identify key genes related to both lipid metabolism disorders and inflammation and to analyze their potential mechanisms. Further, the expression of these key genes was validated using an MAFLD mouse model. Our research provides new scientific insights and potential therapeutic targets for the early identification and treatment of MAFLD.

## 2. Materials and Methods

### 2.1. Data Acquisition

Data analysis in this study followed the workflow outlined in Figure S1. The datasets GSE135251, GSE89632, and GSE186328 were obtained from the GEO database (https://www.ncbi.nlm.nih.gov/geo/, access date: 25 December 2024). GSE135251, designated as the training set, included liver tissue samples from 206 patients with MAFLD and 10 controls. GSE89632, serving as the validation set, comprised samples from 39 patients with MAFLD and 24 controls. GSE186328 provided scRNA-seq data from three MAFLD liver tissue samples and three control samples. Additionally, based on the existing literature, 200 inflammation-related genes (IRGs) and 471 lipid metabolism-related genes (LMRGs) were sourced from the Molecular Signatures Database (MSigDB) (https://www.gsea-msigdb.org/gsea/msigdb, access date: 29 December 2024) for inclusion in this study [8,9].

### 2.2. Differential Expression Analysis

To identify differentially expressed genes (DEGs) between the MAFLD and control samples in the GSE135251 dataset, GEO2R was employed for gene set annotation and differential expression analysis. The criteria for DEG screening were set as |log_2_ Fold Change (FC)| > 1 and *p* < 0.05. The results were visualized with a volcano plot and heatmap, created using ggplot2 (v3.5.1) and ComplexHeatmap (v2.21.1), respectively.

### 2.3. Weighted Gene Co-Expression Network Analysis (WGCNA)

Since there were relatively few IRGs to obtain a meaningful intersection among IRGs, LMRGs, and DEGs, we expanded the IRG gene set using WGCNA. To identify module genes with a relatively high correlation to the IRGs in the training set, WGCNA analysis was performed on GSE135251, with genes divided into multiple modules. The correlation between each module and the IRGs was calculated, and the module with the highest correlation score was selected as the expanded IRG gene set. Specifically, the IRG scores for all samples in GSE135251 were calculated with the GSVA package (v 1.50.0). Significant differences in IRG scores between the MAFLD and control groups were determined via the Wilcoxon test (rstatix v0.7.2). WGCNA (WGCNA v 1.72.5) identified modular genes associated with IRG scores and MAFLD. The top 15,000 genes with the highest variance were selected for further analysis. Furthermore, samples were clustered using GoodSamplesGenes to construct a sample clustering tree and exclude outliers. The soft threshold was 8. Modules of at least 200 genes were formed, with a mergeCutHeight of 0.25. Finally, Pearson correlation analysis identified modules highly correlated with IRG scores and MAFLD (|cor| > 0.30, *p* < 0.05), selecting key module genes.

### 2.4. Identification of Candidate Genes

The intersection of key module genes, DEGs, and LMRGs was identified using the VennDiagram package (v 1.7.3) to find genes associated with both IRGs and LMRGs in MAFLD, labeled as candidate genes.

### 2.5. Discernment of Key Genes Through Machine Learning and Expression Validation

Machine learning facilitated the refinement of key gene selection. Twelve algorithms were applied to the GSE135251 and GSE89632 datasets, generating 113 algorithm combinations. These included Random Forest (randomForestSRC; v 3.2.4), Least Absolute Shrinkage and Selection Operator (LASSO) (glmnet; v 4.1.8), Stepglm (MASS; v 7.3.6), Ridge (glmnet; v 4.1.8), Gradient Boosting Machine (GBM) (gbm; v 2.1.9), Elastic Net (glmnet; v 4.1.8), Linear Discriminant Analysis (LDA) (MASS; v 7.3.6), Support Vector Machine(SVM) (e1071; v 1.7.14), glmboost (mboost; v 2.9.10), XGBoost (mboost; v 2.9.10), plsRglm (plsRglm; v 1.5.1), and Naive Bayes (e1071; v 1.7.14) [10]. Random Forest constructs multiple decision trees and combines their results to enhance model accuracy and stability [11]; LASSO uses L1 regularization for variable selection and shrinkage estimation in high-dimensional data [12]; Stepglm builds models by iteratively adding or removing variables based on statistical tests [12]; Ridge regression applies L2 regularization to address multicollinearity by shrinking coefficients; and GBM constructs a series of decision trees to reduce residuals via gradient descent [13]. Elastic Net combines L1 and L2 regularization to handle high-dimensional data and multicollinearity [14]. LDA maximizes between-class distances and minimizes within-class distances for classification [15]. SVM separates different classes using a hyperplane that maximizes the classification margin [16]; glmboost iteratively optimizes model parameters based on generalized linear models [17]; XGBoost enhances computational efficiency and model performance for large-scale datasets [18]; plsRglm is suitable for high-dimensional data with multicollinearity [19]; and Naive Bayes classifies data using Bayes’ theorem with the assumption of feature independence [20]. Diagnostic models for candidate genes were then developed based on these algorithm combinations in GSE135251 and validated using GSE89632. Model performance was evaluated through Area Under the Curve (AUC) values in both datasets, identifying genes from the best-performing model as potential key genes. All models were all employed with 5-fold cross-validation during the training process to ensure their generalization ability across different data subsets. Furthermore, Receiver Operating Characteristic Curve (ROC) curve analysis, conducted using pROC (v1.18.5), assessed the diagnostic performance of the selected model in detecting MAFLD. Finally, the Wilcoxon test, conducted using rstatix (v 0.7.2), was used to compare the potential key gene expression patterns present between the MAFLD and control groups in both datasets.

### 2.6. Subcellular Localization of the Key Genes

The RCircos package (v 1.2.2) was used to visualize the distribution of key genes across chromosomes, highlighting their structural characteristics. At the same time, their subcellular localization was predicted using the GeneCards database (https://www.genecards.org/, access date: 29 December 2024). Key genes were imported into the BioGPS database (http://biogps.org, access date: 29 December 2024) with a correlation threshold of >0.9, showing the top 5 tissues for each key gene. The relationships between tissue-key genes and subcellular-localization-key genes were visualized using Cytoscape (v 3.9.1).

### 2.7. Analysis of Key Gene-Associated Proteins

Protein-level classification data for key genes were obtained via the PANTHER classification system (https://www.pantherdb.org/, access date: 29 December 2024). Protein interaction networks were constructed using the BioGRID database’s “Networks” module (https://thebiogrid.org/, access date: 29 December 2024), with a concentric circles display. Given that phosphorylation is the most common type of covalent modification in protein post-translational processes, potential phosphorylation sites in the key gene-associated proteins were analyzed using the PhosphoSitePlus database (https://www.phosphosite.org/homeAction.action, access date: 29 December 2024).

### 2.8. Gene Ontology (GO), Kyoto Encyclopedia of Genes and Genomes (KEGG), and Gene Set Enrichment Analysis (GSEA)

GO and KEGG enrichment analyses (clusterProfiler v4.8.3; *p* < 0.05) were conducted alongside GSEA to systematically characterize the biological functions of these genes in MAFLD progression. The Spearman correlation coefficients between each key gene and all other genes in GSE135251 were calculated using the psych package (v 2.4.3) and ranked. The KEGG pathway gene set (c2.cp.kegg.v7.5.1.symbols.gmt) from MSigDB was used as the reference for pathway enrichment analysis. GSEA was performed for each key gene using the clusterProfiler package (v 4.8.3), with thresholds of |Normalized Enrichment Score (NES)| > 1 and *p* < 0.05.

### 2.9. Gene–Gene Interaction (GGI) Network Construction

To elucidate the relationships between key genes and similar genes, as well as their common functions, the GeneMANIA database (http://www.genemania.org/, access date: 29 December 2024) was used to construct a GGI network. Functional correlations within pathways were assessed using functional similarity analysis, and similarity scores between key genes were calculated with the GOsemsim package (v 2.26.1). Higher similarity scores indicated greater functional similarity.

### 2.10. Immune Microenvironment Analysis

The ssGSEA method was employed to evaluate the enrichment scores of 28 immune cell types [21] in the MAFLD and control groups in GSE135251. The Wilcoxon test (*p* < 0.05) was applied to identify immune cell types that showed significant differences between the two groups. To further evaluate the relationships between key genes and differential immune cells, as well as the interrelationships among these immune cells, a Spearman correlation analysis (|r| > 0.3, *p* < 0.05) was performed across all samples in the GSE135251 dataset.

### 2.11. Establishment and Assessment of Nomogram

In GSE135251, a nomogram was developed using the rms package (v 6.5.0) to predict MAFLD occurrence based on the identified key genes. Each key gene was assigned a unique score, with higher cumulative scores indicating a greater risk of MAFLD. The nomogram’s accuracy was verified via a calibration curve generated by the rms package. Moreover, the ROC curve, generated by the pROC package (v 1.18.5), was used to assess the nomogram’s diagnostic value through AUC calculation. Furthermore, decision curve analysis (DCA), conducted using the rmda package (v 1.6), evaluated the nomogram’s clinical utility. A similar approach was applied to construct a nomogram for the validation set GSE89632, followed by calibration curve analysis, ROC analysis, and DCA to validate the diagnostic performance of the model.

### 2.12. scRNA-Seq Data Processing

The single-cell RNA-seq data from GSE186328 were processed using the Seurat package, including quality control (filtering cells with nFeature_RNA between 200 and 5000, and mitochondrial gene percentage less than 5% [percent.mt]) and normalization. Subsequently, the top 2000 genes with the highest variability in expression were identified using the FindVariableFeatures function from the Seurat package. To reduce dimensionality, principal component analysis (PCA) was applied based on these 2000 highly variable genes. The optimal number of principal components (PCs) was determined by visualizing the top 50 PCs using the ElbowPlot function. Next, to identify the number of cell clusters, unsupervised clustering analysis was conducted using the FindNeighbors and FindClusters functions, with a resolution parameter set to 0.4. The clustering results were visualized using uniform manifold approximation and projection (UMAP). Clusters were labeled based on using known marker genes and cell types, utilizing cell markers and the R package xCell (v 1.1.0).

### 2.13. Enrichment Analysis of Key Genes in Different Cell Types

Single-cell analysis was performed to explore the biological pathways of marker genes in various cell types of MAFLD. Marker genes differentially expressed between MAFLD and control samples were identified using the FindAllMarkers function in Seurat with thresholds set as |log_2_FC| > 0.5 and *p* < 0.05. The top 50 genes for each cell type were analyzed using the STRING database (confidence > 0.4), and the top 10 genes were selected via the Maximum Clique Centrality (MCC) algorithm in Cytoscape’s Cytohubba plugin. KEGG enrichment analysis was conducted for the 50 top-ranked genes, applying a significance cutoff of *p* < 0.05.

### 2.14. Cellular Communication Analysis and Expression of Key Genes in scRNA-Seq Data

The CellChat package (v 1.6.1) was used to infer the interaction dynamics among annotated cells in GSE186328, comparing the MAFLD and control groups with reference to the CellChatDB.human database. Ligand–receptor interactions and cell type distributions between the two groups were visualized using heatmaps generated with ggplot2 (version 3.5.1). Percentage differences in cell types were analyzed by Fisher’s test (*p* < 0.05). Additionally, UMAP and violin plots were generated with Seurat (version 5.1.0) and ggplot2 (version 3.5.1) to examine the distribution and expression levels of key genes.

### 2.15. Animals and Diet

Male C57BL/6 mice (n = 10, 8-week-old) from the Institute of Laboratory Animal Science (CAMS & PUMC, Beijing, China) were housed in SPF-grade conditions with a controlled temperature, humidity, and 12 h light/dark cycle. After a 14-day acclimatization period, the mice were randomly assigned to the following two experimental groups (five mice for each group): (1) control group (standard chow diet, H10010, Research Diets; 3.85 kcal/g, 10% fat/20% protein/70% carbohydrate) and (2) MAFLD model group (high-fat diet [HFD], H10060, Research Diets; 5.24 kcal/g, 60% fat/20% protein/20% carbohydrate). The dietary intervention was maintained for 8 weeks to induce MAFLD. Animal experiments were conducted with ethical approval (IRB2025-DW-25, 17 March 2025) from Tianjin Medical University General Hospital.

### 2.16. Real-Time Polymerase Chain Reaction (PCR) Analysis

The total RNA of the liver was isolated with the TRIzol kit (Ambion, Austin, TX, USA, 15596-018CN), and then cDNA was synthesized with the TIANScript RT kit (TIANGEN, Inc., Beijing, China). Real-time PCR was carried out following established protocols [22]. All mRNA measurements were normalized to GAPDH (endogenous control) and analyzed through the 2^−ΔΔCt^ comparative threshold cycle method. The primer sequences for the target genes are provided in Table S1.

### 2.17. Activator Prediction and Molecular Docking

The activators targeting key genes were identified through the Drug Signatures Database (DsigDB) (https://maayanlab.cloud/DsigDB, access date: 29 December 2024). The activator with the highest interaction scores for all key genes was selected as a candidate. Three-dimensional structural data were downloaded from PubChem (https://pubchem.ncbi.nlm.nih.gov/, access date: 29 December 2024), while corresponding protein–ligand complex structures (including crystallographic data) were acquired from the Protein Data Bank (https://www.rcsb.org/, access date: 29 December 2024). Molecular docking of the activators with proteins corresponding to each key gene was performed using PyMOL software (v 3.0.3).

### 2.18. Statistical Analysis

All statistical evaluations were performed using R statistical software (v 4.2.2), with between-group comparisons analyzed through the non-parametric Wilcoxon rank-sum test. Real-time PCR data analysis involved paired two-sided Student’s *t*-tests for Ct value comparisons, executed in GraphPad Prism (v10.4.0). Statistical significance was defined as probability values below the 0.05 threshold.

## 3. Results

### 3.1. Identification of Candidate Genes with Both Lipid Metabolism Disorders and Inflammation

**Figure 1:**
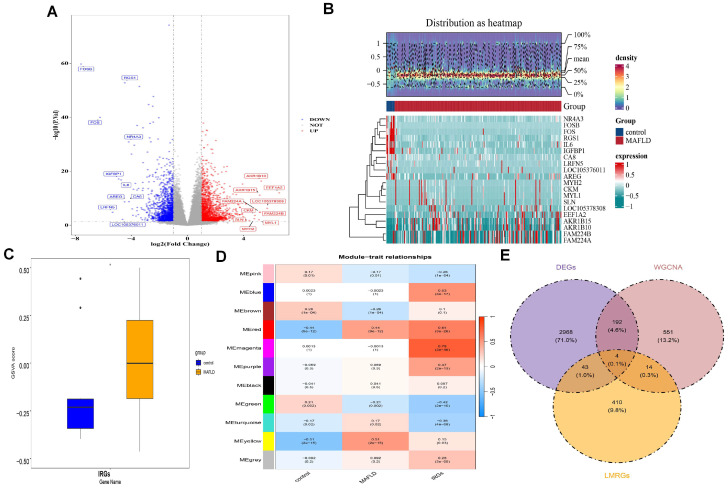
Identification of candidate genes associated with both lipid metabolism disorders and inflammation. (**A**) Volcano plot showing DEGs with the criteria of |logFC| > 1 and *p*-value < 0.05. Red and blue circles indicate upregulated and downregulated DEGs, respectively. The dashed line indicates the position where |log_2_ FC| = 1. (**B**) Heatmap of the expression profiles of the top 10 upregulated and downregulated DEGs. (**C**) Inflammation-related gene (IRG) scores were significantly higher in the MAFLD group compared to the control group in GSE135251. * *p* < 0.05. (**D**) The MEred module of the WGCNA network constructed from GSE135251 shows a strong correlation with IRG scores. (**E**) Intersection analysis of 3207 DEGs, 761 genes in the MEred module, and 471 LMRGs identified 4 candidate genes (FADS1, FADS2, GLB1, and PNPLA3).

Differential expression analysis identified 3207 DEGs between the MAFLD and control groups in GSE135251, with 1690 upregulated and 1517 downregulated genes in MAFLD. The top 10 most significantly altered genes are shown in a volcano plot (Figure 1A), and their expression profiles are displayed in a heatmap (Figure 1B). Notably, in the GSE135251 dataset, the MAFLD cohort exhibited significantly higher IRG scores compared to controls (*p* < 0.05) (Figure 1C). Afterwards, a WGCNA network was constructed using all GSE135251 samples, with no outliers detected (Figure S2A). A soft threshold of eight was chosen, yielding a signed R^2^ of 0.85 and mean connectivity close to zero (Figure S2B). Hierarchical clustering revealed 11 co-expression modules (Figure S2C). The MEred module, strongly correlated with IRG scores (cor = 0.64, *p* < 0.001) and MAFLD (cor = 0.44, *p* < 0.001), emerged as a key module containing 761 genes (Figure 1D). The intersection of 3207 DEGs, 761 key module genes, and 471 LMRGs identified 4 candidate genes (FADS1, FADS2, GLB1, and PNPLA3) (Figure 1E).

### 3.2. Four Key Genes Associated with MAFLD Were Identified Using Machine Learning

**Figure 2:**
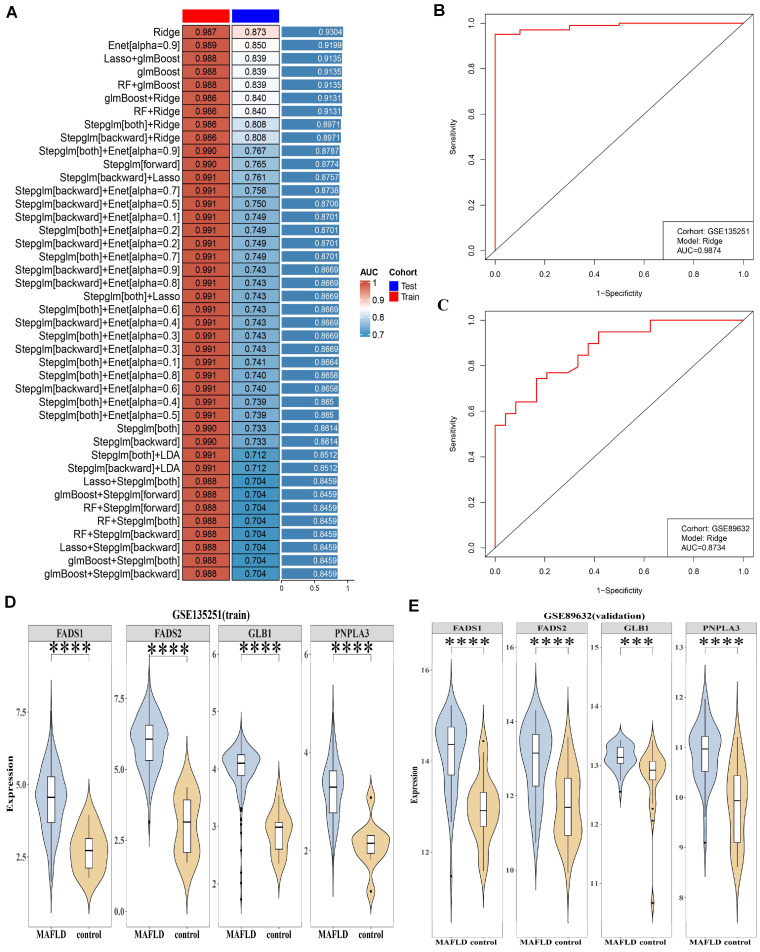
FADS1, FADS2, GLB1, and PNPLA3 were identified as key genes for MAFLD using machine learning. (**A**) AUC values for 113 prediction models from GSE135251 (training cohort) and GSE89632 (test cohort) (**B**) AUC values for the Ridge model in the ROC curve for GSE135251:0.9874. (**C**) AUC values for the Ridge model in the ROC curve for GSE89632: 0.8734. The red line demonstrates the actual performance of the classification model, with curves positioned higher or to the left indicating superior classification capabilities. (**D**,**E**) Expressions levels of FADS1, FADS2, GLB1, and PNPLA3 were significantly higher in the MAFLD group compared to the control group in both GSE135251 and GSE89632. *** *p* < 0.001; **** *p* < 0.0001.

A machine learning model was developed to identify the key genes associated with both IRGs and LMRGs in MAFLD. The AUC values of 113 prediction models from GSE135251 and GSE89632 are shown in Figure 2A. The Ridge model was selected as the optimal model, achieving an average AUC of 0.9304 across both datasets. It identified FADS1, FADS2, GLB1, and PNPLA3 as potential key genes. The AUC values for the Ridge model in the ROC curves were 0.9874 for GSE135251 and 0.8734 for GSE89632 (Figure 2B,C). Of particular note, all four genes exhibited marked upregulation in the MAFLD cohort relative to healthy controls, a pattern consistently observed in both the GSE135251 and GSE89632 datasets (*p* < 0.05) (Figure 2D,E). These results confirm the stability and reliability of FADS1, FADS2, GLB1, and PNPLA3 as key genes.

### 3.3. The Exploration of the Functions of the Four Key Genes

A detailed analysis of the four key genes was conducted to reveal their locations and functions. FADS1 and FADS2 are located on chromosome 11, GLB1 is on chromosome 3, and PNPLA3 is on chromosome 22 (Figure S3A). GLB1 is present in the Golgi apparatus, lysosome, and extracellular space (Figure S3B). FADS1, FADS2, and PNPLA3 are localized to the endoplasmic reticulum, with FADS1 and FADS2 also found in the plasma membrane. FADS1 and FADS2 co-distribute in the adrenal gland, B lymphoblasts, and adipocytes (Figure S3C), while FADS2 and PNPLA3 co-distribute in the liver. GLB1 is widely distributed in tissues such as the lung.

Next, at the protein level, FADS1 and FADS2 are oxidoreductases, GLB1 is a galactosidase, and PNPLA3 is a phospholipase (Table S2). FADS1 interacts with 96 proteins, FADS2 interacts with 82 proteins, GLB1 interacts with 123 proteins, and PNPLA3 interacts with 8 proteins (Figure S3D–G). In addition, FADS1 and FADS2 are mainly modified by ubiquitylation (Figure S3H,I). GLB1 undergoes phosphorylation and ubiquitylation (Figure S3J), while PNPLA3 undergoes phosphorylation and acetylation (Figure S3K).

**Figure 3:**
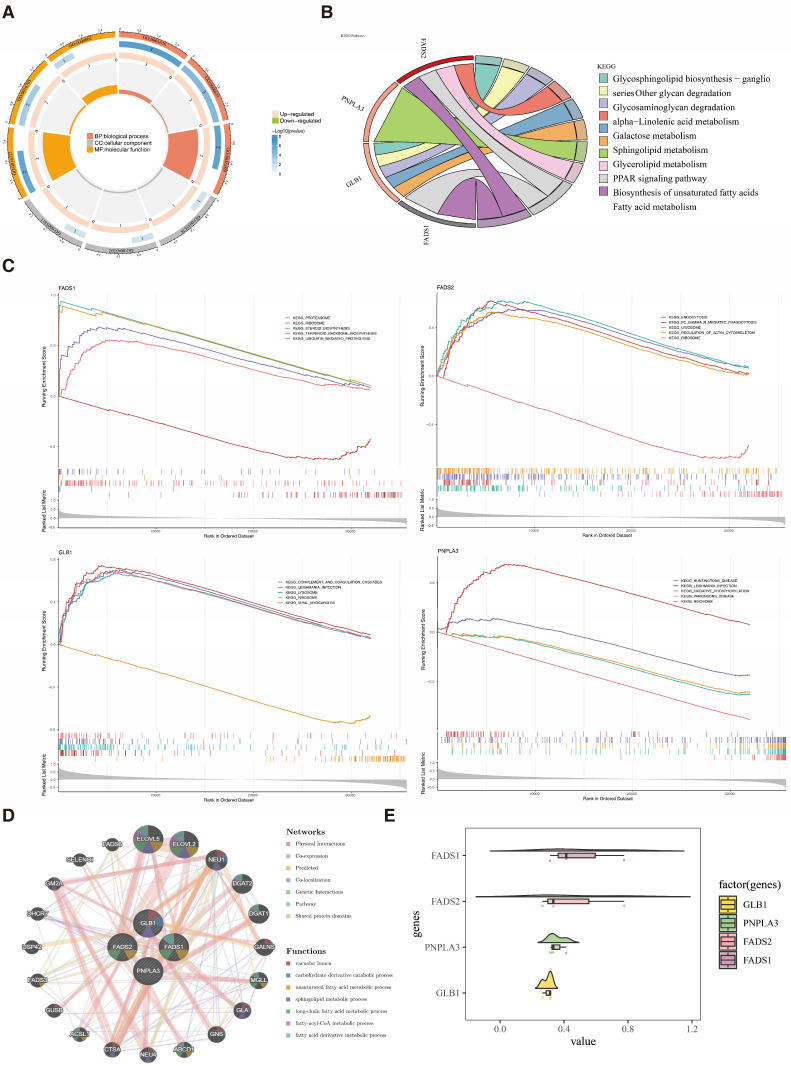
Exploration of the functions of the four key genes. (**A**) GO enrichment analysis revealed significant enrichment in 91 GO terms related to the four key genes. (**B**) KEGG analysis showed significant enrichment of the four key genes in fatty acid metabolism and related signaling pathways. (**C**) GSEA analysis diagram of the four key genes. (**D**) GGI network related to the four key genes was constructed. (**E**) FADS1 had the highest functional similarity score among the four key genes.

GO enrichment analysis demonstrated significant associations of the four key genes with 91 functional terms (*p* < 0.05), distributed across 65 biological processes (BPs), 8 cellular components (CCs), and 18 molecular functions (MFs). The leading BPs included “long-chain fatty acid metabolic process”, “fatty acid metabolic process”, and “alpha-linolenic acid metabolic process” (Figure 3A). The top CCs were “azurophil granule lumen”, “lysosomal lumen”, and “lipid droplet”. The top MFs involved “fatty-acyl-CoA binding” and “oxidoreductase activity”. These annotations suggest that the four key genes are crucial in fatty acid synthesis, catabolism, and energy conversion. Moreover, KEGG analysis further revealed significant associations (*p* < 0.05) between the four key genes and 10 biological pathways, most notably the PPAR signaling pathway and unsaturated fatty acid biosynthesis (Figure 3B), highlighting their importance in fatty acid metabolism and related signaling. Moreover, during the GSEA analysis, the “ribosome” pathway emerged as a common enriched pathway among the top five pathways (|NES| > 1, *p* < 0.05) for all four key genes (Figure 3C). These results highlight that the key genes could be functionally involved in ribosomal functions or protein synthesis regulation.

Finally, the GGI network related to the four key genes was constructed, which included 24 genes, with the 4 key genes surrounded by 20 genes (Figure 3D). In this network, FADS1 and ELOVL2 shared functions such as fatty acid derivative metabolism. Interestingly, FADS1 had the highest functional similarity score among the four key genes (Figure 3E), suggesting potential overlaps in its biological functions or network interactions with other key genes.

### 3.4. The Correlation Between Key Genes and Differential Immune Cells

**Figure 4:**
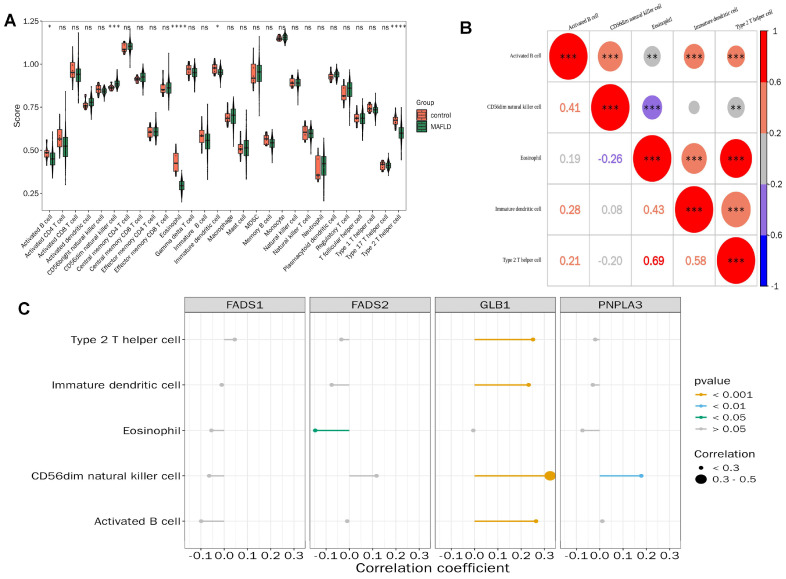
Correlation between key genes and differential immune cells. (**A**) Comparative analysis of immune cell enrichment scores between the MAFLD and control groups in GSE135251; (**B**) correlation between different immune cells; and (**C**) correlation analysis between differential immune cells and key genes. ns indicates *p* > 0.05; * *p* < 0.05; ** *p* < 0.01; *** *p* < 0.001; **** *p* < 0.0001.

The immune microenvironment was further investigated in the MAFLD and control groups from GSE135251. Significant differences (*p* < 0.05) were observed in the enrichment scores of the following five specific immune cell types: activated B cells, CD56dim natural killer (NK) cells, eosinophils, immature dendritic cells, and Th2 cells (Figure 4A). Notably, type2 T helper cells demonstrated the highest correlation with eosinophils (correlation coefficient = 0.69, *p* < 0.001) (Figure 4B). Additionally, a significant positive correlation was observed between GLB1 expression and CD56dim NK cell abundance (correlation coefficient = 0.33, *p* < 0.05) (Figure 4C).

### 3.5. Nomogram Using the Key Genes Showed Good Performance in Evaluating MAFLD

**Figure 5:**
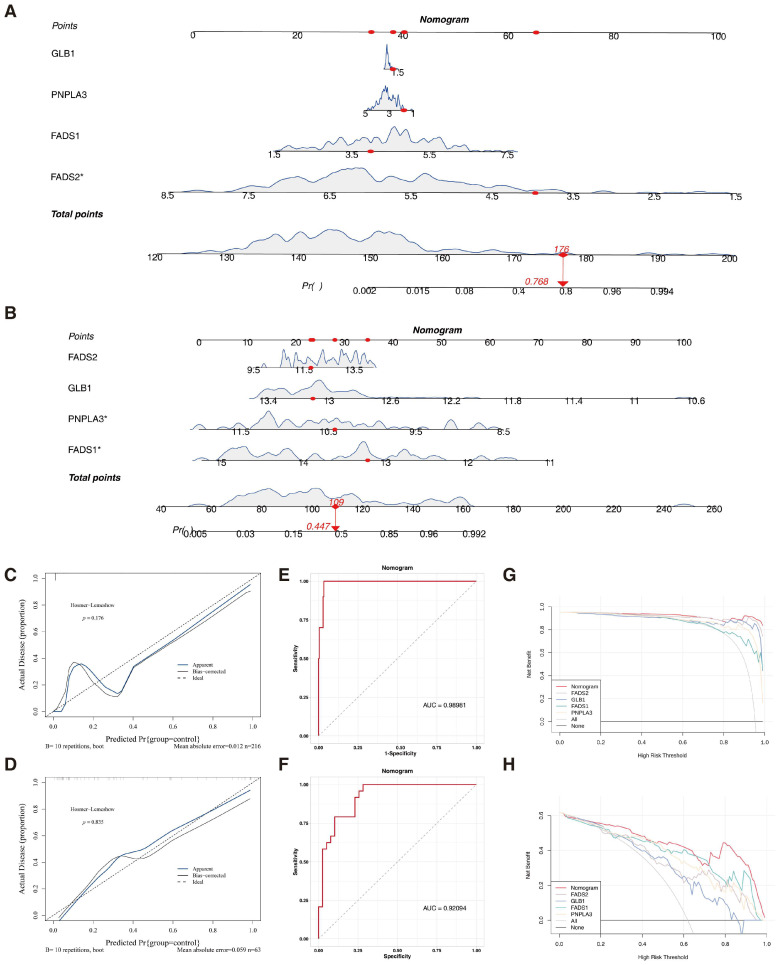
Nomogram constructed using key genes demonstrated strong performance in evaluating MAFLD. (**A**) The nomogram constructed in GSE135251. Red dots indicate the “score markers” for each variable in the nomogram scoring system. Genes marked with an asterisk contribute more reliably to the predictive model. (**B**) The nomogram constructed in GSE89632. (**C**) The calibration curve slopes for GSE135251. (**D**) The calibration curve slopes for GSE89632. (**E**) The AUC values in the ROC curves for GSE135251. (**F**) The AUC values in the ROC curves for GSE89632. The red line demonstrates the actual performance of the classification model, with curves positioned higher or to the left indicating superior classification capabilities. (**G**) The DCA of the nomogram in GSE135251. (**H**) The DCA of the nomogram in GSE89632.

A nomogram was constructed to evaluate the diagnostic value of the key genes for MAFLD. The model showed that a higher total score correlated with an increased probability of developing MAFLD both in GSE135251 and GSE89632 (Figure 5A,B). The calibration curve slopes for GSE135251 (*p* = 0.176) and GSE89632 (*p* = 0.835), which were close to one, indicated minimal diagnostic errors (Figure 5C,D). The AUC values in the ROC curves for GSE135251 and GSE89632 were 0.98981 and 0.9204, respectively, suggesting a high predictive accuracy (Figure 5E,F). In addition, the DCA showed that the nomogram offered a greater net benefit compared to individual key genes (Figure 5G,H), suggesting its potential to improve early MAFLD diagnosis.

### 3.6. The Acquisition of Seven Annotated Cell Types in scRNA-Seq Data

To further analyze the expression and distribution of the four key genes at the single-cell level, scRNA-seq data from GSE186328 were utilized. Initially, the dataset contained 47,724 cells and 21,181 genes. After applying quality control filters, the cell count decreased to 22,461, while the gene count remained constant at 21,181 (Figure S4A,B). Following processing, 2000 highly variable genes were identified (Figure S4C), with the top 10 being HLA-DRA, CD74, CST3, HLA-DRB1, C1QB, C1QC, CCL20, HLA-DQA1, LYZ, and VCAN. In addition, PCA analysis showed a uniform distribution of single-cell samples between the MAFLD and control groups, with no batch effects (Figure S4D,E). The top 30 PCs were selected for further analysis (Figure S4F). Subsequently, UMAP clustering delineated 22 distinct cell clusters (Figure S4G). Finally, marker gene analysis identified the following seven cell types: B cells, endothelial cells, hematopoietic stem cells (HSCs), monocytes, neutrophils, NK cells, and T cells (Figure S4H). The expressions of the first three marker genes for each cell type were visualized in bubble and UMAP plots (Figure S4I,J–P).

### 3.7. Enrichment Analysis of Differentially Expressed Marker Genes in Seven Annotated Cell Types

A comprehensive enrichment analysis was conducted for the seven annotated cell types. The UMAP plots for B cells, endothelial cells, HSCs, monocytes, neutrophils, NK cells, and T cells are shown in Figure S5A–G. Interactions among the top 10 differentially expressed marker genes in these seven cell types from both the MAFLD and control groups are illustrated in Figure S4H–N. Notably, the top 50 differentially expressed marker genes in endothelial cells, neutrophils, NK cells, and T cells were co-enriched in the “cytokine–cytokine receptor interaction” pathway, while the top 50 marker genes in B cells, HSCs, and monocytes were primarily enriched in the “B-cell receptor signaling pathway”, “neutrophil extracellular trap formation”, and “phagocytosis”, respectively. These annotated cell types are crucial in immune responses through specific signaling pathways and functions, including pathogen recognition and clearance, antibody production, and modulation of inflammatory responses.

### 3.8. Cellular Communication Analysis in scRNA-Seq Data

Cellular communication analysis revealed complex interactions among the seven annotated cell types. In the MAFLD group, monocytes, neutrophils, and B cells exhibited frequent and intense interactions (Figure S6A,B). In contrast, the control group showed frequent and intense interactions among monocytes, neutrophils, HSCs, NK cells, and T cells (Figure S6C,D). In the MAFLD group, monocytes most frequently interacted with HSCs and B cells, whereas in the control group, T cells predominantly interacted with monocytes and HSCs (Figure S6E,F). Notably, significant differences were observed in the distribution of HSCs, T cells, and NK cells between the MAFLD and control groups. Specifically, the MAFLD group had a 0.88% increase in HSCs and a 12.39% increase in T cells compared to the control group, while the proportion of NK cells was 11.8% lower (Figure S6G).

### 3.9. The Distribution of Four Key Genes in the Seven Annotated Cell Types

**Figure 6:**
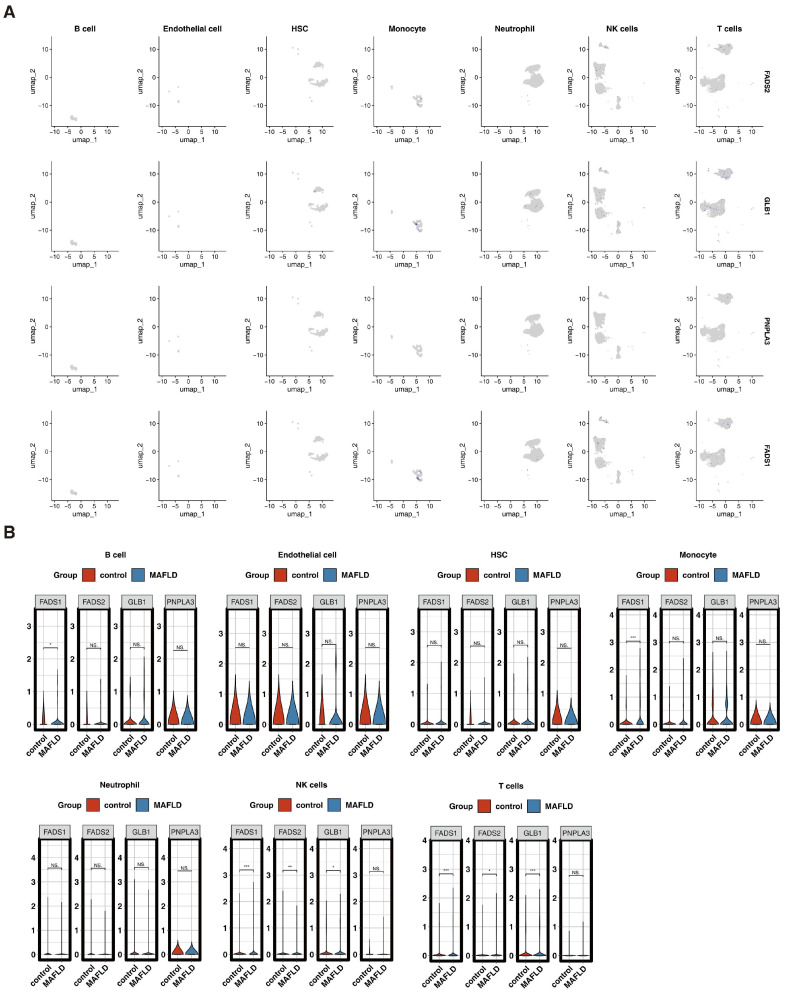
Distribution of the four key genes in different cell types in GSE186328. (**A**) UMAP plots showing the distribution of four key genes across seven annotated cell types. (**B**) Expression differences of the four key genes between MAFLD and control samples in various cell types. ns indicates *p* > 0.05; * *p* < 0.05; ** *p* < 0.01; *** *p* < 0.001. Red represents the control group, blue represents the MAFLD group.

FADS1 and FADS2 were predominantly expressed in monocytes, NK cells, and T cells, while GLB1 showed a higher concentration in NK cells and T cells (Figure 6A). PNPLA3 expression was low across all annotated cell types. Notably, FADS1, FADS2, and GLB1 exhibited significant differences in expression levels (*p* < 0.05) between patients with MAFLD and controls in both NK cells and T cells (Figure 6B).

### 3.10. The Expression of the Four Key Genes in the MAFLD Mouse Model

**Figure 7:**
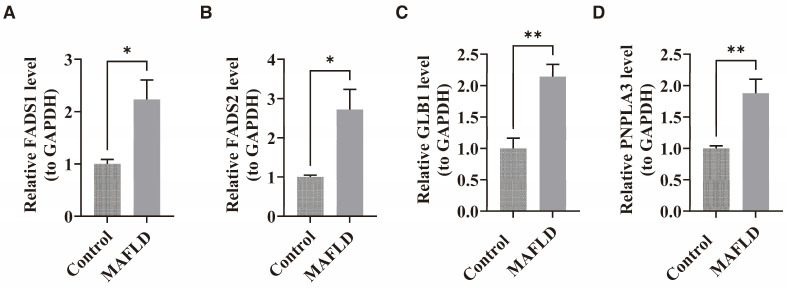
Verification of the four key genes expression in mice via real-time PCR. (**A**) Relative mRNA expression of FADS1 in MAFLD and control mice. (**B**) Relative mRNA expression of FADS2 in MAFLD and control mice. (**C**) Relative mRNA expression of GLB1 in MAFLD and control mice. (**D**) Relative mRNA expression of PNPLA3 in MAFLD and control mice. All data are presented as mean ± SEM. Control: n = 5, MAFLD: n = 5. * *p* < 0.05, ** *p* < 0.01.

The mRNA levels of FADS1, FADS2, GLB1, and PNPLA3 were evaluated through real-time PCR in MAFLD and control mice. Compared to the controls, the mRNA expression of these four genes was significantly higher in the MAFLD mice (*p* < 0.05) (Figure 7A–D). These results are consistent with the transcriptomic patterns observed in the GSE135251 and GSE89632 datasets, validating the bioinformatics predictions and supporting the reliability of the study’s conclusions.

### 3.11. Estradiol Was Identified as an Activator Targeting All Four Key Genes

**Figure 8:**
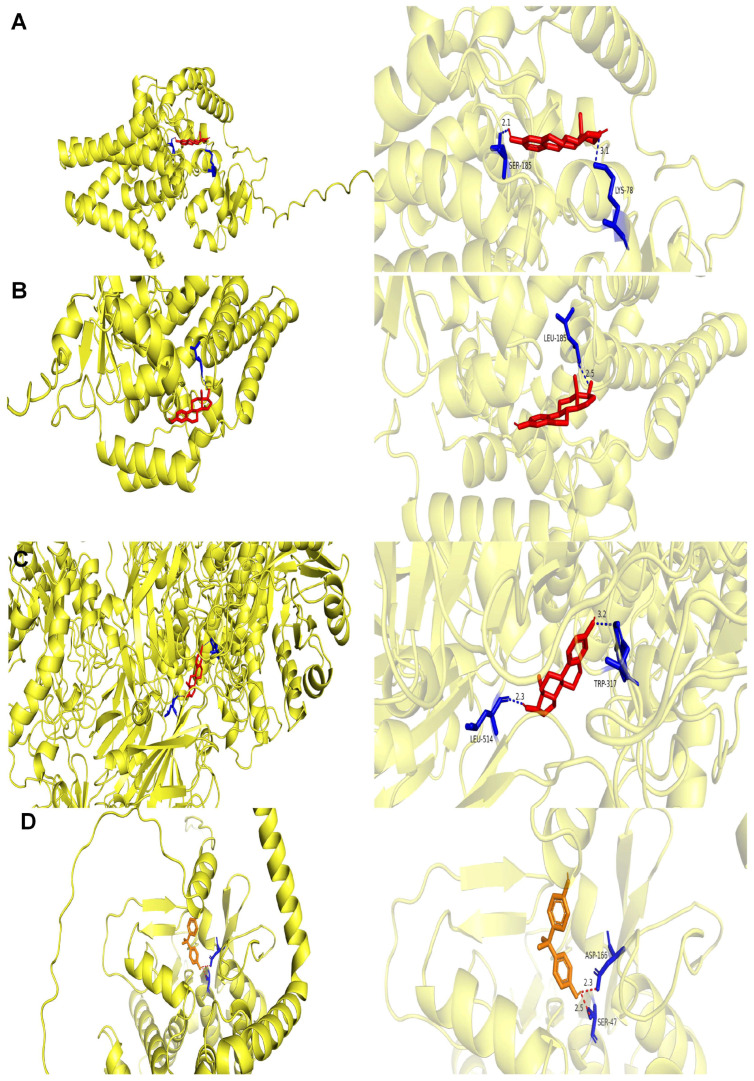
Estradiol identified as an activator targeting all four key genes. (**A**) Schematic diagram of the docking between FADS1 and estradiol, showing binding sites SER-185 and LYS-78 with hydrogen bond lengths of 2.1 and 3.1 Å. (**B**) Schematic diagram of the docking between FADS2 and estradiol, showing binding site LEU-185 with a hydrogen bond length of 2.5 Å. (**C**) Schematic diagram of the docking between GLB1 and estradiol, showing binding sites LEU-514 and TRP-317 with hydrogen bond lengths of 2.3 and 3.2 Å. (**D**) Schematic diagram of the docking between PNPLA3 and estradiol, showing site SER-453 with a hydrogen bond length of 2.2 Å. Light yellow represents the three-dimensional structure of the protein; red indicates ligands, small molecules, or key functional domains bound to the protein; blue denotes amino acid residues or structural fragments interacting with the ligands (red regions); orange signifies another structural element with specific functions.

Computational screening for potential activators using DsigDB identified compounds targeting the four key genes. This in silico approach predicted 53 potential activators for FADS1 (17 FDA-approved), 37 for FADS2 (14 FDA-approved), 9 for GLB1 (5 FDA-approved), and 181 for PNPLA3 (75 FDA-approved). Among the FDA-approved drugs predicted to target these genes, estradiol emerged as a candidate of interest due to its computational association with all four targets, suggesting a potential for multi-target engagement. To preliminarily explore this possibility, molecular docking studies were performed. The calculated binding free energies of estradiol to PNPLA3 (−9.6 kcal/mol), FADS2 (−8.6 kcal/mol), GLB1 (−8.5 kcal/mol), and FADS1 (−7.7 kcal/mol) suggested favorable binding interactions, exceeding the commonly used threshold of ≤−5 kcal/mol. Moreover, hydrogen bond interactions with critical residues were observed for all targets (Figure 8A–D; Table S3 for bond distances and residues). It is important to note that these findings are derived solely from computational prediction, requiring rigorous experimental validation.

## 4. Discussion

Lipid metabolism disorders and inflammation are established critical factors driving the progression of MAFLD. However, until now, there has been a lack of data integrating both of them to investigate the key genes implicated in MAFLD progression.

In this study, we identified four key genes, including FADS1, FADS2, GLB1, and PNPLA3, associated with both metabolic disorders and inflammation in MAFLD through bioinformatics methods. We also observed a consistent upregulation of these four key genes in the MAFLD mouse model.

The study highlighted four key genes, two of which, FADS1 and FADS2, belong to the fatty acid desaturase gene family. These two genes are functionally linked, encoding enzymes responsible for introducing double bonds into long-chain polyunsaturated fatty acids (PUFAs) through desaturation [23]. FADS1 encodes delta-5 desaturase, while FADS2 encodes delta-6 desaturase. These enzymes are essential for converting dietary essential fatty acids into longer-chain PUFAs, such as eicosatetraenoic acid (EPA) and arachidonic acid (AA), which play important roles in inflammation and metabolic processes [24]. Our results are consistent with earlier studies indicating that FADS1/FADS2 polymorphisms are associated with altered PUFA profiles and increase the risk of MAFLD [25]. Moreover, elevated expressions of FADS1/FADS2 in MAFLD may promote excessive ω-6 PUFA synthesis, exacerbating inflammation and lipotoxicity. This mechanism is consistent with our observation of increased oxidative stress and immune activation in MAFLD models.

GLB1 encodes β-galactosidase, a lysosomal enzyme involved in the degradation of glycoprotein and glycolipid. While its role in MAFLD is less well understood, GLB1 dysfunction has been linked to lysosomal storage disorders and impaired autophagy [26,27]. Our study demonstrated that GLB1 is upregulated in MAFLD and co-enriched with FADS1/FADS2 in the ribosomal pathway. This suggests a potential interaction between lysosomal activity, ribosome biogenesis, and lipid metabolism. Further studies are needed to clarify whether GLB1 overexpression is a compensatory response to Endoplasmic Reticulum (ER) stress or contributes to hepatocyte injury through impaired lysosomal function.

PNPLA3 is a well-established genetic determinant of MAFLD, with the I148M variant strongly associated with liver steatosis and fibrosis [28,29]. PNPLA3 regulates lipid droplet remodeling and triglyceride hydrolysis [30]. Our findings confirm previous reports of PNPLA3 upregulation in MAFLD, likely due to lipid overload and ER stress. Notably, the co-enrichment of PNPLA3 in the ribosomal pathway suggests its potential involvement in stress-induced protein synthesis, which represents a novel area for further investigation.

In this study, we also explored the functions of the four key genes using GO, KEGG, and GSEA enrichment analyses. The four key genes were co-enriched in the “ribosome” pathway, indicating ribosomal dysfunction as a potential driver of MAFLD. Ribosomes, which are essential for protein synthesis, are known to be implicated in metabolic stress, the unfolded protein response (UPR), and inflammation [31,32]. While the link between ribosomal dysfunction and MAFLD has not been explicitly described in previous studies, chronic lipid overload in MAFLD induces ER stress, activating UPR pathways that demand increased ribosomal activity [33]. Additionally, mitochondrial dysfunction plays a pivotal role in the development and progression of MAFLD [34]. As ribosomal dysfunction may impact the synthesis of mitochondrial proteins, it could exacerbate mitochondrial dysfunction, contributing to the pathophysiology of MAFLD [35]. Future studies may investigate whether targeting ribosomal pathways can mitigate the progression of MAFLD.

The immune microenvironment in MAFLD is highly complex, involving intricate interactions among various immune cells, such as B cells, T cells, macrophages, and dendritic cells. These cells form a sophisticated regulatory network through the secretion of cytokines and chemokines, collectively driving liver inflammation [36]. In this study, we analyzed the characteristics of the immune microenvironment of MAFLD and explored the correlations between key genes and various immune cells, providing preliminary insights into the potential role of CD56dim NK cells in MAFLD. CD56dim NK cells are cytotoxic and contribute to liver inflammation both through direct hepatocyte lysis and the secretion of pro-inflammatory cytokines like IFN-γ [37]. Our analysis revealed significant changes in the immune cell population in patients with MAFLD, particularly an increase in the number of CD56dim NK cells. Additionally, a strong correlation was found between GLB1 and CD56dim NK cells, which may be linked to lysosomal dysfunction or impaired autophagy. These pathological processes could lead to the accumulation of undigested substrates within cells, triggering the release of damage-associated molecular patterns (DAMPs) and activating CD56dim NK cells [38]. Our findings provide a new perspective on the immune pathology of MAFLD. Future studies will investigate GLB1’s role in NK cell function, offering a foundation for targeted MAFLD therapies.

In addition, we analyzed the expression and distribution of the four key genes at the single-cell level. Single-cell profiling revealed cell-type-specific expressions of FADS1, FADS2, and GLB1, with notable differences between NK and T cell populations. In MAFLD, NK cells showed upregulated FADS1/FADS2, which may enhance their pro-inflammatory activity via AA-derived eicosanoids [39]. T cells exhibited increased GLB1 expression, which could impair immune tolerance through lysosomal dysfunction. These findings are consistent with previous studies highlighting the role of NK and T cells in MAFLD-related inflammation and fibrosis [40,41]. Additionally, the reduced proportion of NK cell proportions in MAFLD compared to the control group may indicate exhaustion or recruitment to damaged hepatocytes, a phenomenon observed in advanced fibrosis [42].

In this study, we identified estradiol as a multi-target activator of the four key genes and highlighted its potential therapeutic role in MAFLD. Previous studies have demonstrated that estrogen exerts a protective effect against the development of MAFLD, primarily through its interactions with estrogen receptor-α (ER-α) [43]. The key genes we identified in this study, including FADS1, FADS2, and PNPLA3, have also been reported to be involved in this process. For instance, ERα can bind to specific estrogen response elements within the promoter regions of target genes, including FADS1, FADS2, and PNPLA3, thereby modulating their transcription [44,45]. Although our molecular docking results indicated strong binding between estradiol and GLB1, limited research directly links GLB1 to estradiol. Perhaps more studies related to this interaction could be conducted in the future. Additionally, given the side effects associated with high-dose estradiol, such as an increased risk of thrombosis, endocrine disruption, and limitations in male patients [46], future research may focus on evaluating estrogen analogues as potential MAFLD interventions.

The model developed in this study showed a strong performance (AUC > 0.8) in both the GSE135251 and GSE89632 datasets. Nonetheless, we acknowledge the risk of overfitting due to high-dimensional genomic data and addressed this by using a final Ridge regression model with L2 regularization, which reduced multicollinearity and overfitting by limiting coefficient magnitude. Furthermore, model performance was evaluated on an independent external validation set (GSE89632), where the Ridge model achieved an AUC of 0.8734, confirming its generalization ability. Additionally, real-time PCR validated the expression patterns of key genes, consistent with bioinformatics predictions, supporting the reliability of the gene selection. However, models based on public datasets still need validation in prospective large-scale multicenter studies to assess their clinical applicability.

This study, using bioinformatics methods, identified four key genes integrating both lipid metabolism disorders and inflammation in MAFLD. Further verification in the MAFLD mouse model confirmed the consistent upregulation of these gene expressions, providing new insights for elucidating the pathogenesis of MAFLD. However, this study is limited by its clinical relevance and external validation. Our findings rely on public datasets and a mouse model. While key gene expression was verified in animal models, the lack of validation in human cohorts remains a significant gap. Future studies should include larger multicenter cohorts to assess the diagnostic and prognostic value of these genes across diverse patient populations. Although the key-gene-based nomogram performed well in predicting MAFLD, its clinical utility requires further real-world evaluation. DCA demonstrated its greater net benefit compared to individual genes, but further validation and optimization are necessary for clinical application. Finally, the sample size of the single-cell RNA sequencing data used in this study is relatively small, and the obtained results are only preliminary exploratory findings. Increasing the sample size and incorporating more single-cell datasets will help us gain a more comprehensive understanding of the cell heterogeneity and intercellular interactions in MAFLD.

## 5. Conclusions

This study identifies FADS1, FADS2, GLB1, and PNPLA3 as key genes integrating both lipid metabolism disorders and inflammation in MAFLD, with potential diagnostic implications. Their enrichment in ribosomal pathways and association with immune dysregulation, such as CD56dim NK cells, reveals novel mechanisms underlying the disease. Estradiol targets all four genes, suggesting its therapeutic potential. Although this study highlights the clinical potential of these genes, further research is required to validate their translational value for MAFLD diagnosis and treatment.

## Data Availability

The original data used in this project can be downloaded from the public database GEO (https://www.ncbi.nlm.nih.gov/geo/, access date: 25 December 2024). The data analyzed during the in vitro experiments are available from the corresponding author upon reasonable request.

## Supplementary Materials

The following supporting information can be downloaded at: https://www.mdpi.com/article/10.3390/biomedicines13092211/s1, Figure S1: Flowchart of this study; Figure S2: A WGCNA network was constructed to expand the IRG gene set; Figure S3: The localization, function and post-translational modifications of the four key genes; Figure S4: Acquisition of 7 annotated cell types in the scRNA-seq dataset GSE186328; Figure S5 Enrichment analysis of marker genes of the 7 annotated cell types in GSE186328; Figure S6 Cellular communication analysis in GSE186328; Table S1: The primer sequences of target genes; Table S2: Protein classification information for FADS1, FADS2, GLB1, and PNPLA3; Table S3: Hydrogen bond interactions between estradiol and target proteins in molecular docking studies.
