# Supplementary material for: Identification of Key Genes Related to Both Lipid Metabolism Disorders and Inflammation in MAFLD
Source: Biomedicines. 2025 Sep 9;13(9):2211. doi: 10.3390/biomedicines13092211 (PMC12467344; doi:10.3390/biomedicines13092211)
Supplement: Supplementary file 1 [file biomedicines-13-02211-s001.zip › biomedicines-3748359-supplementary.pdf]

Supplementary Information

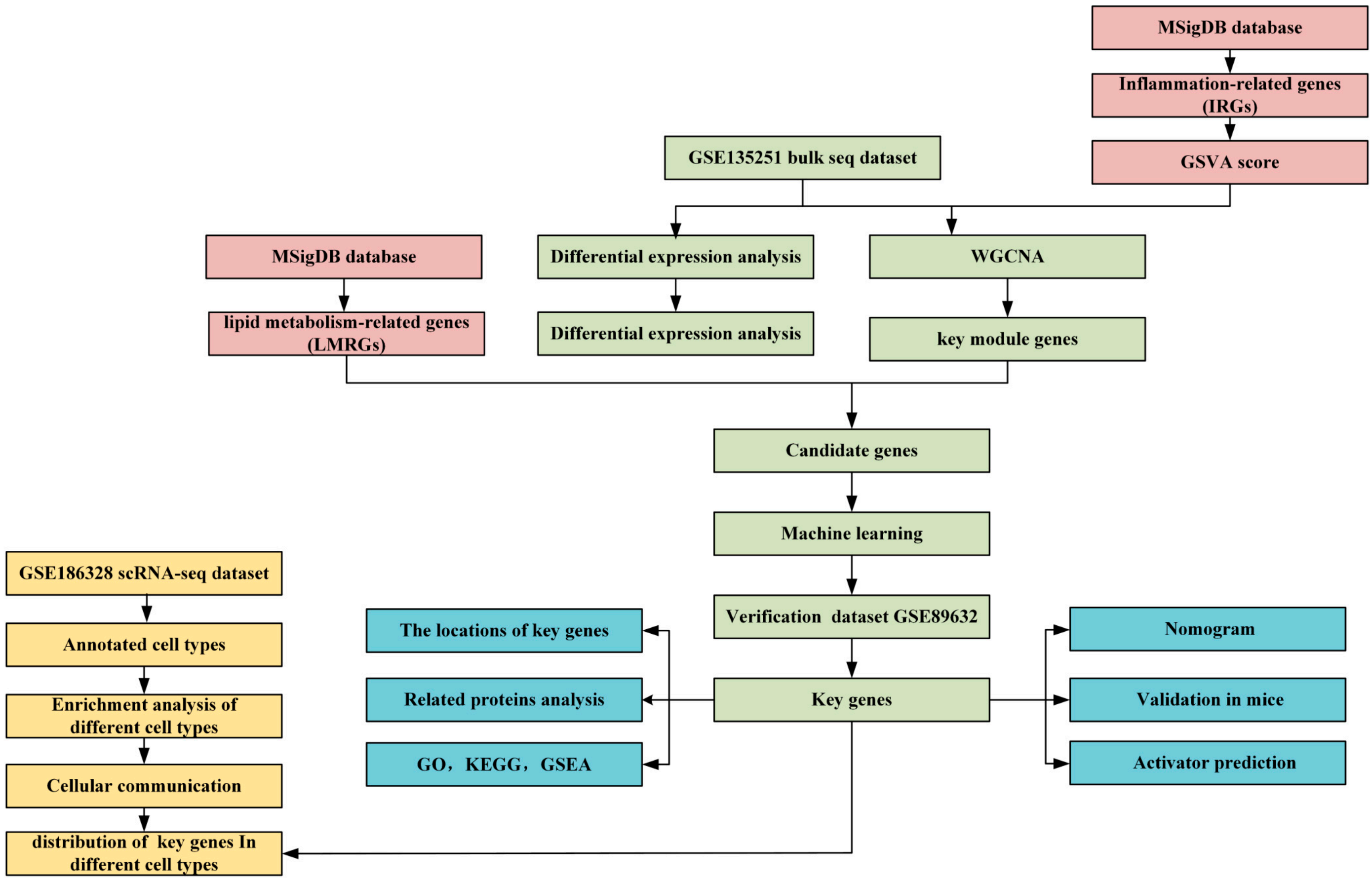

Figure S1. Flowchart of this study.

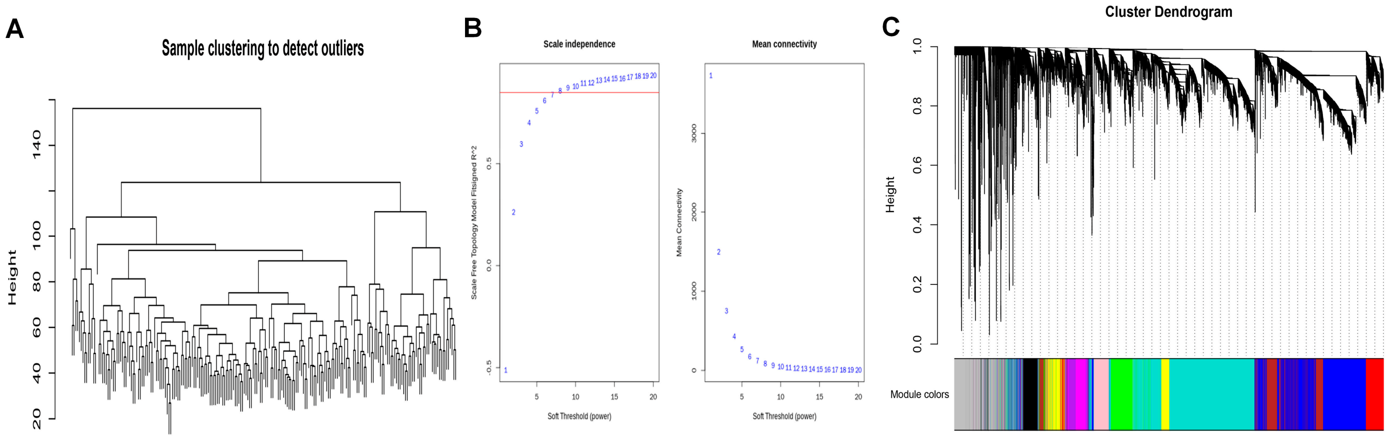

Figure S2. A WGCNA network was constructed to expand the IRG gene set (**A**) A WGCNA network was constructed using all GSE135251 samples, with no outliers detected. (**B**) Analysis of the scale-free index and the mean connectivity for various soft-threshold powers. (**C**) Displays the merged modules under the cluster tree. WGCNA: Weighted gene co-expression network analysis; IRG: inflammation-related genes

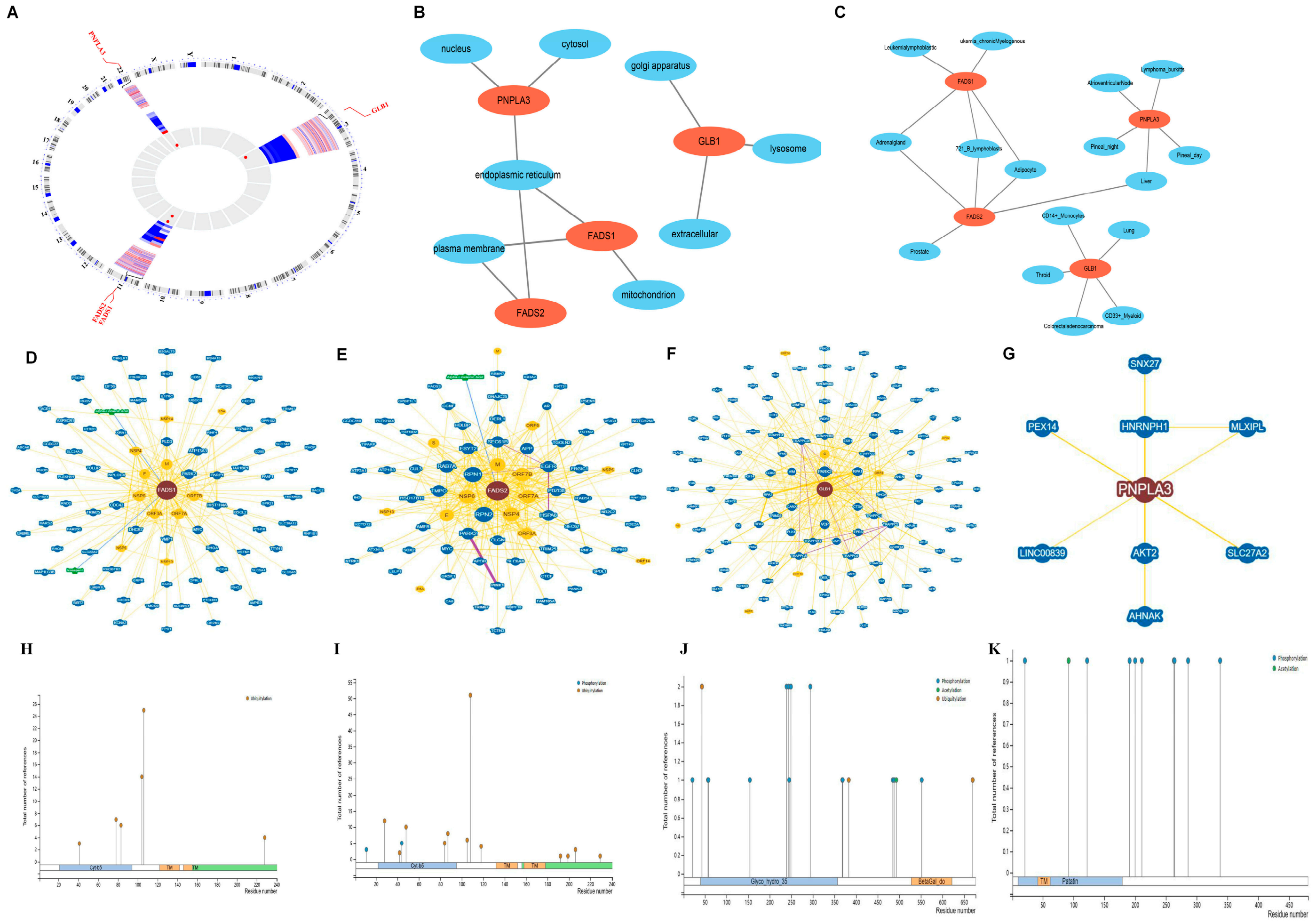

Figure S3. The localization, function, and post-translational modifications of the four key genes (**A**) The localization of key genes on chromosomes. (**B**) The subcellular distribution of the four key genes. (**C**) The distribution of the four key genes in organs and tissues. (**D–G**) The interaction between the proteins coded by the four key genes and other proteins. (**H–K**) The potential phosphorylation sites of the four key genes at the protein level.

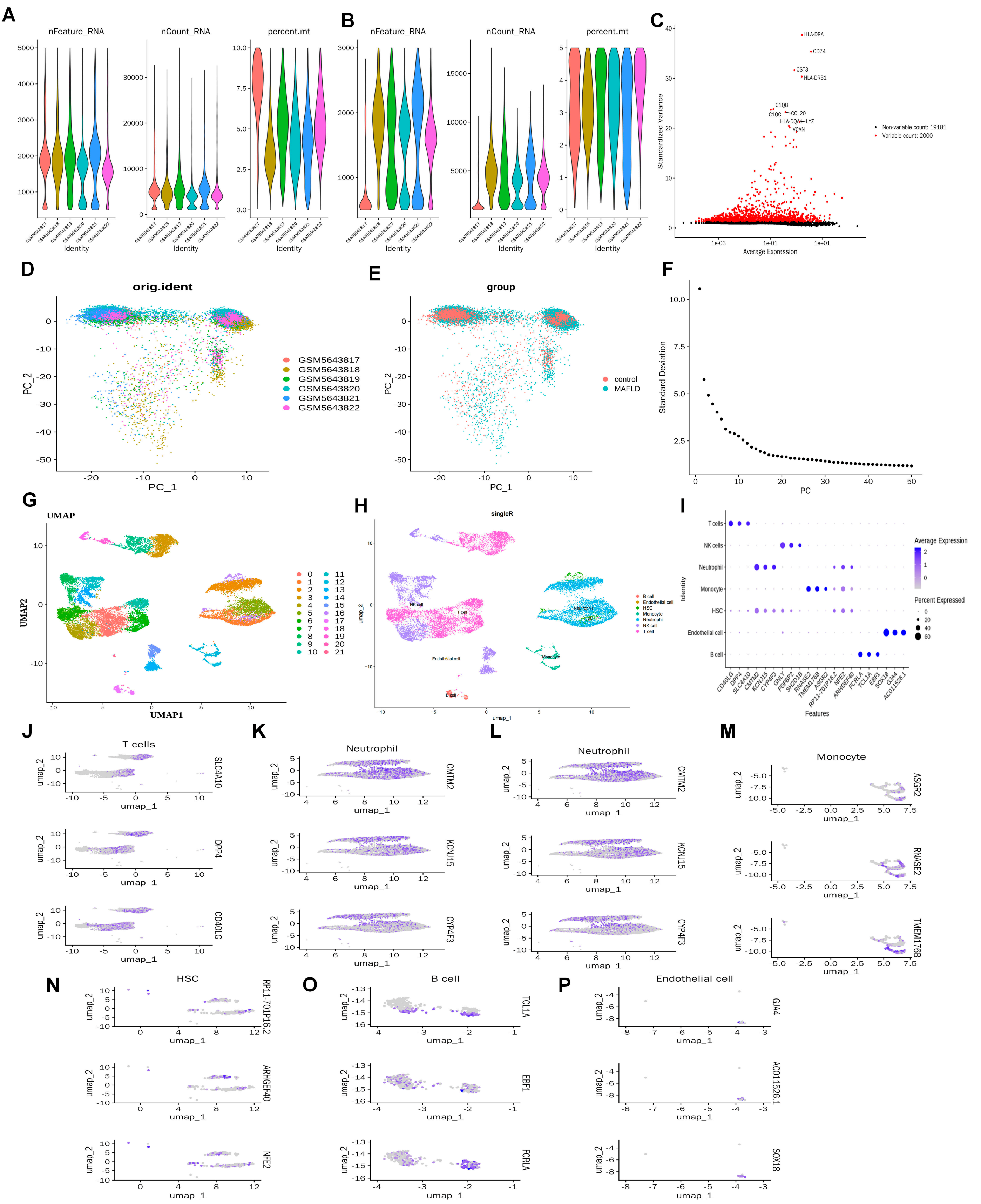

Figure S4. Acquisition of 7 annotated cell types in the scRNA-seq dataset GSE186328 (**A**) The distribution maps of nFeature_RNA, nCount_RNA, and percent.mt before quality control. (**B**) The distribution maps of nFeature_RNA, nCount_RNA, and percent.mt after quality control. (**C**) 2,000 highly variable genes were identified with top 10 hypervariable genes labeled. (**D**) DCA analysis showed the distribution of cells in each sample. (**E**) DCA analysis showed the distribution of cells between

MAFLD and control. (**F**) The top 50 PCs were visualized using the ElbowPlot function. (**G**) UMAP clustering delineated 22 distinct cell clusters. (**H**) Marker genes identified 7 cell types: B cells, endothelial cells, HSCs, monocytes, neutrophils, NK cells, and T cells. (**I**) Bubble plot of marker gene expression in different cell types. (**J–P**) The distribution of marker genes in different cells. PC: principal components; DCA: decision curve analysis; UMAP: uniform manifold approximation and projection; MAFLD: Metabolic Associated Fatty Liver Disease

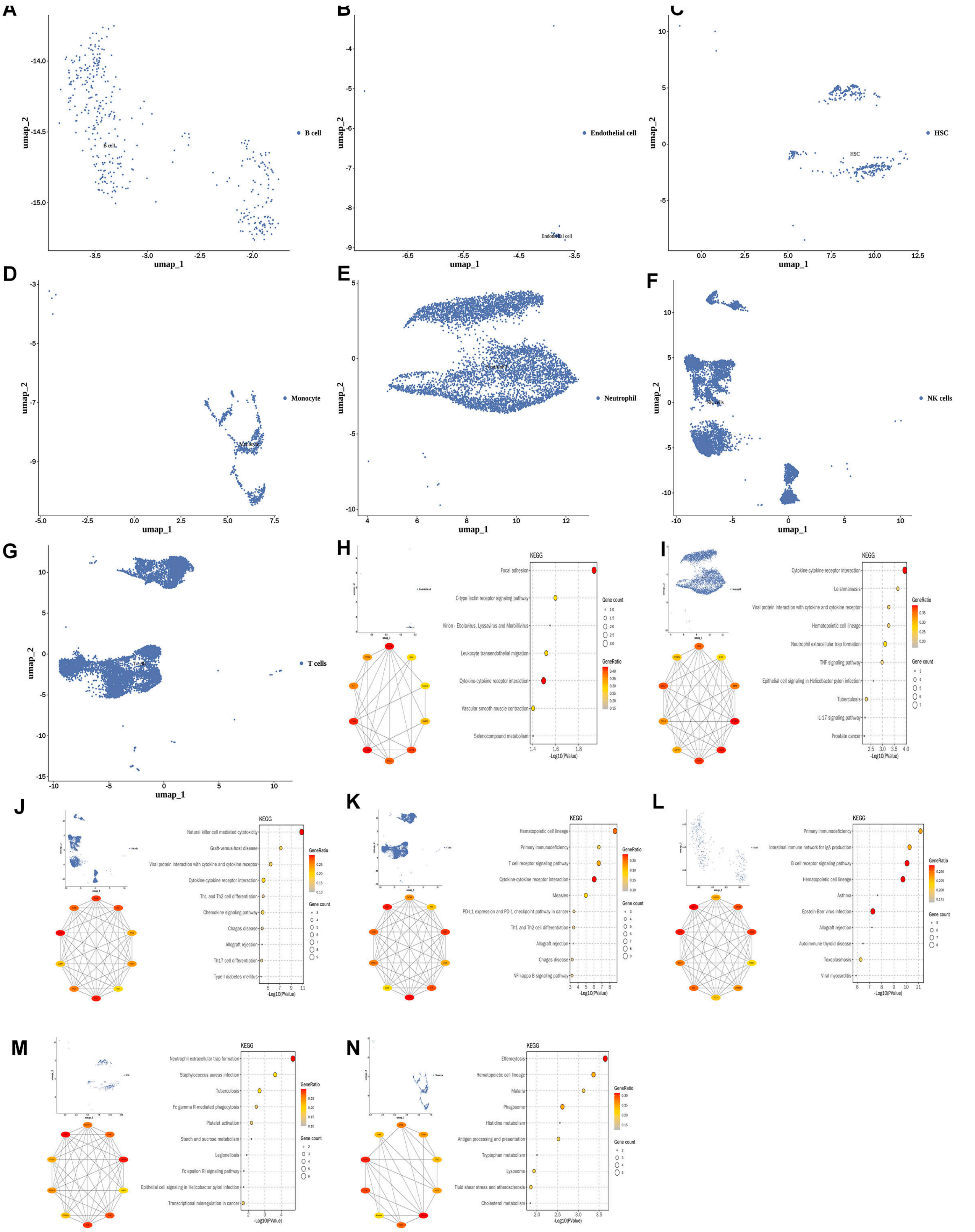

Figure S5. Enrichment analysis of marker genes of the 7 annotated cell types in GSE186328. (**A–G**) The 7 annotated cell types were presented with UMAP plots. (**H–N**) Enrichment analysis of the marker genes in the 7 annotated cell types. UMAP: uniform manifold approximation and projection.

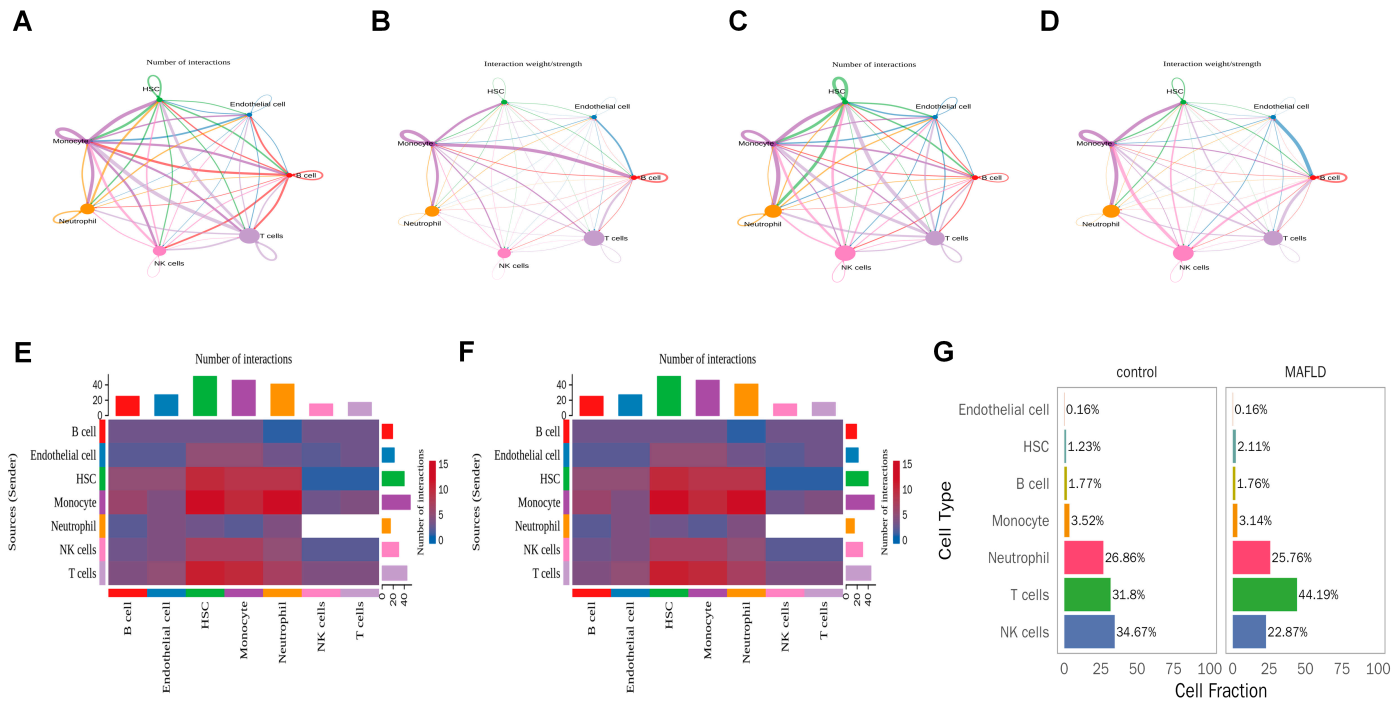

Figure S6. Cellular communication analysis in GSE186328. (**A**) The quantity of cell communication among different cell types in the MAFLD group. (**B**) The intensity of cell communication among different cell types in the MAFLD group. (**C**) The quantity of cell communication among different cell types in the control group. (**D**) The intensity of cell communication among different cell types in the control group. (**E**) The correlation of the quantity and intensity of intercellular interactions in the MAFLD group. (**F**) The correlation of the quantity and intensity of intercellular interactions in the MAFLD group. (**G**) The differences in the distribution of annotated cell types between the MAFLD and control groups. MAFLD: Metabolic Associated Fatty Liver Disease.

Table S1. The primer sequences of target genes.

Primer		Sequences
FADS1	F	CAACATCAGCGACTTCAGCC
FADS1	R	GCGCTTTATTCTTGGTGGGT
FADS2	F	CACAGCCTTTGTCCTCGCTA
FADS2	R	GGCTGAGGCACCCTTTAAGT
GLB1	F	TCCGGATACCCCGCTTCTAC
GLB1	R	CACGGTCCCCAGAAAACTCA
PNPLA3	F	CCCTCTCGGCCGTATAATGG
PNPLA3	R	CCCGTCTCTGATGCACTTGT
GAPDH	F	TGTGTCCGTCGTGGATCTGA
GAPDH	R	GAGTTGCTGTTGAAGTCGCA

Table S2. Protein classification information for FADS1, FADS2, GLB1, and PNPLA3.

ID	Gene name	Gene ID	PANTHER family/subfamily	PANTHER protein class
GLB1	Beta-galactosidase	HUMAN|HGNC=4298|UniProtKB=P16278	BETA-GALACTOSIDASE (PTHR23421:SF172)	galactosidase
FADS1	Acyl-CoA (8-3)-desaturase	HUMAN|HGNC=3574|UniProtKB=O60427	ACYL-COA (8-3)-DESATURASE (PTHR19353:SF57)	oxidoreductase
FADS2	Acyl-CoA 6-desaturase	HUMAN|HGNC=3575|UniProtKB=O95864	ACYL-COA 6-DESATURASE (PTHR19353:SF12)	oxidoreductase
PNPLA3	1-acylglycerol-3-phosphate O-acyltransferase PNPLA3	HUMAN|HGNC=18590|UniProtKB=Q9NST1	1-ACYLGLYCEROL-3-PHOSPHATE O-ACYLTRANSFERASE PNPLA3 (PTHR12406:SF22)	phospholipase

Table S3. Hydrogen bond interactions between estradiol and target proteins in molecular docking studies.

Gene	Drug	Residue(s)	Bond Distance(Å)	Binding Free Energy (kcal/mol)
FADS1	Estradiol	SER-185, LYS-78	2.1, 3.1	−7.7
FADS2	Estradiol	LEU-185	2.5	−8.6
GLB1	Estradiol	LEU-514, TRP-317	2.3, 3.2	−8.5
PNPLA3	Estradiol	SER-453	2.2	−9.6
